# RNAi Mediated Tiam1 Gene Knockdown Inhibits Invasion of Retinoblastoma

**DOI:** 10.1371/journal.pone.0070422

**Published:** 2013-08-07

**Authors:** Nithya Subramanian, Saranya Navaneethakrishnan, Jyotirmay Biswas, Rupinder K. Kanwar, Jagat R. Kanwar, Subramanian Krishnakumar

**Affiliations:** 1 Larsen and Toubro Department of Ocular Pathology, Vision Research Foundation, Sankara Nethralaya, Chennai, India; 2 Nanomedicine Laboratory of Immunology and Molecular Biomedical Research (N-LIMBR), School of Medicine (SoM), Molecular and Medical Research (MMR) Strategic Research Centre, Faculty of Health, Deakin University, Geelong Technology Precinct (GTP), Geelong, Victoria, Australia; H. Lee Moffitt Cancer Center & Research Institute, United States of America

## Abstract

T lymphoma invasion and metastasis protein (Tiam1) is up-regulated in variety of cancers and its expression level is related to metastatic potential of the type of cancer. Earlier, Tiam1 was shown to be overexpressed in retinoblastoma (RB) and we hypothesized that it was involved in invasiveness of RB. This was tested by silencing Tiam1 in RB cell lines (Y79 and Weri-Rb1) using siRNA pool, targeting different regions of Tiam1 mRNA. The cDNA microarray of Tiam1 silenced cells showed gene regulations altered by Tiam1 were predominantly on the actin cytoskeleton interacting proteins, apoptotic initiators and tumorogenic potential targets. The silenced phenotype resulted in decreased growth and increased apoptosis with non-invasive characteristics. Transfection of full length and N-terminal truncated construct (C1199) clearly revealed membrane localization of Tiam1 and not in the case of C580 construct. F-actin staining showed the interaction of Tiam1 with actin in the membrane edges that leads to ruffling, and also imparts varying invasive potential to the cell. The results obtained from our study show for the first time that Tiam1 modulates the cell invasion, mediated by actin cytoskeleton remodeling in RB.

## Introduction

Retinoblastoma (RB) is an intraocular tumor of childhood. As per International Retinoblastoma Staging Working Group (IRSWG) most of the RB tumors are found to have massive choroidal, optic nerve, and anterior segment invasion [1]. The risk factors include choroidal invasion >3 mm (CI>3 mm), post laminar and surgical end of optic nerve invasion. Understanding the molecular regulation of tumor cell invasion and apoptosis helps in identifying new therapeutic targets.

T lymphoma invasion and metastasis protein (Tiam1) was first identified as an invasion and metastasis inducing gene using T lymphoma cells by proviral tagging and *in vitro* selection for invasiveness [2], [3]. Tiam1 is a guanine nucleotide exchange factor (GEF) that mediates the specific activation of Rac1 [4], [5], [6]. Small guanine triphosphate (GTP) binding proteins belonging to Ras superfamily act as molecular switches for activation of cellular activities such as signal transduction, actin cytoskeleton remodeling, microtubule stabilization, centrosome reorganization and intracellular trafficking [7], [8], [9], [10]. Aberrations or mutations of these proteins lead to malignancy of the cell. In response to extracellular signals, GEFs play a major role by catalyzing the activation of GTP-binding proteins by dissociation of guanosine diphosphate bound to it. RhoA, Rac1 and Cdc42 are key proteins of Rho family that depends on GEFs for their acitivation [11], [12].

Tiam1 has been linked with cancer progression and having growth promoting functions based on the tumor type. Overexpression of N-terminus truncated Tiam1 is found to impart oncogenic activity in NIH 3T3 cells [13], [14]. Similarly, mutations in Tiam1 gene are able to transform NIH 3T3 cells [15]. Oncogenic potential of Tiam1 was found to be present in various tumors with respect to the tumor grade and stage. The over expression of Tiam1 in breast carcinoma, nasopharyngeal carcinoma, hepatocellular carcinoma, renal cell carcinoma, retinoblastoma, colorectal carcinoma, lung and prostate cancer has been previously reported [16], [17], [18], [19], [20], [21], [22]. Tiam1 is negatively correlated in case of renal carcinoma, where it inhibits invasion by promoting E-cadherin mediated adhesion [15].

**Figure 1:**
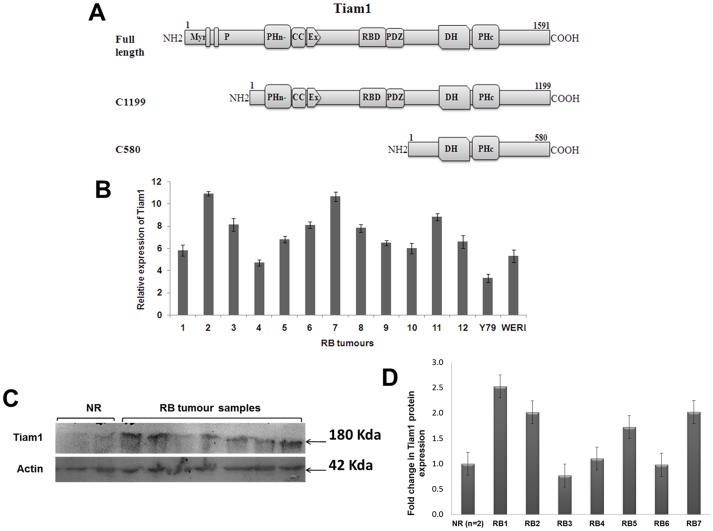
Schematic representation of Tiam1 constructs and expression level of Tiam1 in RB tumors compared to normal retina. **A.** Full length Tiam1 consists of 1591 aminoacids. Tiam1 has several specific domains such as, Myr: Myristoylation site; P: PEST sequences; PHn: N-terminal PH domain; CC: Coiled-coil region; Ex: Extended structure; RBD: Ras-binding domain; PDZ: PSD-95/DlgA/ZO-1 domain; DH: Dbl homology domain and PHc: C-terminal PH domain. Deletion of N-terminal myr and P sites leaves the active C1199 form of Tiam1 in truncated constructs. C580 contains only DH and C-terminal PH domain. **B.** Fold difference in the expression of Tiam1 mRNA in RB tumors versus normal retina by qPCR analysis. The relative fold change (log2 ratio) in Tiam1 mRNA expression is calculated by normalizing with β2-microglobulin using normal retina as control sample. **C.** Western blot analysis of Tiam1 protein expression in RB tumors and normal retina shows Tiam1 at 180 kda and β-actin at 42 Kda. **D**. Densitometry analysis of the Tiam1 protein expression in RB tumors vs normal retina (n = 2). RB1 to RB7 invidividual expression normalized with averaged NR is represented.

Tiam1 contains consensus myristoylation sequence at the amino terminus, two PEST sequences, a Ras binding domain (RBD), PSD-95/DlgA/ZO-1 domain (PDZ), two pleckstrin homology (PH) domains and DH domain [23], [24], [25] (Figure 1A). The PH domain present in carboxy terminal next to DH domain is similar in all other GEFs. The DHR domain is important for the protein-protein interaction [26]. Presence of Tiam1 on the membrane surface is accomplished through the N-terminal PH domain, and not by the c-terminal PH domain or DHR domain. Using truncated constructs of Tiam1, localization of Tiam1 in the membrane is shown to be necessary for the membrane ruffling [24], [27], [28].

Earlier, we showed the expression of Rho, Rac, Cdc42 and Tiam1 in RB. Additional studies on E-Cadherin and N-Cadherin has helped us to correlate the expression levels of these antigens with respect to the function they mediate in the RB tumor progression. Especially the expression of Tiam1 was positively correlated with the invasive potential of the tumors [18]. The functional relevance behind this overexpression of Tiam1 in tumorogenesis and invasion of RB is not yet elucidated. In the current study, we analyzed the effect of Tiam1 on cell proliferation and invasion using RB cell lines, Y79 and Weri-Rb1. Additionally, we addressed the effects of the truncated constructs with respect to subcellular localization and F-actin based interaction and invasiveness attributed to the RB cell lines.

## Materials and Methods

### Cell culture

Human RB cell lines Y79 and Weri-Rb1 were obtained from the cell bank, RIKEN BioResource Center (Ibaraki, Japan). Roswell Park Memorial Institute (RPMI) 1640 media, Heat- inactivated Fetal Bovine Serum (FBS), Poly-L-Lysine (PLL) were purchased from Sigma Aldrich (St. Louis, MO). The cells were cultured in RPMI 1640 medium, supplemented with 10% fetal bovine serum (FBS) (Gibco, Invitrogen, India) along with 1% Pen-strep (Hi-Media, Mumbai, India). The cells were maintained in 5% CO_2_ incubator at 37°C.

### Sample collection and Ethics statement

Tumors samples were collected from enucleated eyeballs of RB patients as a part of treatment with written consent from patient/guardian and utilized for research purpose anonymously. Retina samples were collected from normal cadaveric donor eyeballs from C U shah eye bank, Medical Research Foundation, Sankara Nethralaya, India. This work was approved by Vision Research Foundation ethics committee and done at Vision Research Foundation, Sankara Nethralaya (Ethical clearance no: 136-2008-P).

### Transfection and RNA interference of Tiam1

Full-length and N-terminal truncated C1199 and C580 Tiam1 containing a hemagglutinin tag at the 3′ end, cloned in the eukaryotic expression vector pUTSV1 (Eurogentec, Belgium) were procured from John G Collard, Netherlands Cancer Institute [13]. Tiam1 constructs were transiently transfected in Y79 and Weri-Rb1 cell lines with LipofectAMINE 2000 (Invitrogen) as prescribed in manufacture's protocol. For Tiam1 silencing, three pre-designed short interfering RNA (siRNA) sequences targeting different regions of Tiam1 mRNA were purchased from Qiagen, Valencia, USA. The siRNA sequences are as follows, GGCGAGCUUUAAGAAGAAATT (sense) and UUUCUUCUUAAAGCUCGCCGT (antisense) for Tiam1_1 (T1), CAUGUAGAGCACGAGUUUUTT (sense) and AAAACUCGUGCUCUACAUGTT (antisense) for Tiam1_5 (T5), GGUUCUGUCUGCCCAAUAATT (sense) and UUAUUGGGCAGACAGAACCAG (antisense) for Tiam1_6 (T6). Briefly, the cells were transfected with 200 nM of pooled siRNA sequences by using LipofectAMINE 2000. Scrambled siRNA was used as a negative control in all experiments. The cells were collected after 48 hrs for further experiments.

### Total RNA isolation and Quantitative PCR

The total mRNA was isolated from transfected cells and fresh RB tumors by RNAeasy kit (Qiagen, Valencia, USA) and Trizol respectively. 1 µg of total RNA was transcribed into cDNA using oligo-dT and random hexamers. The quantitative PCR was performed in Applied Biosystem 7500 by using Sybr-green (Thermoscientific, Mumbai, India). The primer sequences for Tiam1, 18S and β2M were as follows, FP: 5′AAGACGTACTCAGGCCATGTCC-3′and RP: 5′-GACCCAAATGTCGCAGTCAG-3′ for Tiam1, FP: 5′- AACCCGTTGAACCCCATT-3′ and RP: 5′-CCATCCAATCGGTAGTAG-3′ for 18S, and FP: 5′-TATCCAGCGTACTCCAAAGA-3′ and RP: 5′- GACAAGTCTGAATGCTCCAC –3′ for β2M. Comparative quantification (normal retina vs primary RB tumors or untreated cells vs Tiam1 siRNA treated cells) was determined using the formula 2^−ΔΔCt^ and relative expression values normalized to the 18S or β2M endogenous control were used for plotting. Experiments were performed in triplicate for the same samples.

### Western Blotting

Transfected cells and RB tumors were collected and washed with 1X PBS followed by cell lysis in RIPA buffer (containing 50 mM Tris-HCl (pH 7.4), 150 mM NaCl, 1 mM PMSF, 1 mM EDTA, 1%Triton X-100, 1% Sodium deoxycholate, 0.1% SDS and Protease inhibitor cocktail, Sigma). 50–100 µg of protein lysate was resolved on 8% SDS Polyacrylamide gel and then electroblotted onto nitrocellulose membrane at 100V for 90 min. The membrane was blocked in 5% skimmed milk, further incubated with primary anti-Tiam1 polyclonal antibody (raised in rabbit, C-16, Santacruz) in 1∶100 dilutions and anti-β actin monoclonal antibody (raised in mouse, Sigma) in 1∶2000 at 4°C for overnight. The membrane was washed thrice with TBST (1 M Tris-HCl pH-7.6 containing 0.1% Tween-20 and 0.8% NaCl) and incubated with HRP-conjugated secondary antibody at 1∶2500 dilution for 2 hrs. The membrane was detected by supersignal west femto maximum sensitivity substrate (Thermoscientific, Rockford, USA). The blots are representative of three experiments.

### Immunofluorescence

For immunofluorescence, cells were seeded on Poly-L-Lysine coated cover slips in 24-well plate. After 48 hrs of transfection, cells were fixed with 4% paraformaldehyde, permeabilized with 0.1% Triton X-100 and blocked with 5% BSA in PBS for 30 mins. The cells were incubated with polyclonal anti-Tiam1 antibody (1∶25) for overnight at 4°C. After PBS wash cells were incubated with FITC conjugated anti-rabbit secondary antibody (1∶500) for 2 hrs at room temperature then incubated with TRITC conjugated phalloidin at 1∶300 dilution (Sigma Aldrich, St. Louis, MO) for 30 min whereas DAPI was used as a nuclear stain. The cover slips were mounted and viewed under Axio Observer fluorescence microscope (Zeiss, Germany).

### Flow cytometry analysis of apoptosis

For the apoptosis assay, Annexin V kit from BD biosciences(San Diego, CA) was used. Briefly, cells were washed with ice cold 1X PBS twice and resuspended in 1X binding buffer and incubated with annexin V-FITC and Propidium iodide for 15 mins, followed by flow cytometry. Experiments were performed thrice individually.

### Cell proliferation assay

3-(4, 5-Dimethylthiazol-2-yl)-2, 5-diphenyltetrazolium bromide (MTT) assay was performed to evaluate the percentage viability of silenced cells. Transfected cells were incubated with 100 μl of media containing 10 μl of MTT (5 mg/ml) and incubated for 4 hrs at 37°C. Then media with MTT was removed and 100 μl of DMSO was added to each well the absorbance was measured at 570 nm. All experiments were performed in triplicate.

### cDNA microarray

Whole genome microarray was performed in Tiam1 siRNA treated Y79 cells along with untransfected Y79 cells. The experiment was performed in triplicates. In brief, 500 ng of total RNA was used for cDNA synthesis, followed by amplification/labeling using TotalPrep RNA Amplification kit (Ambion Inc., Austin, TX) to synthesize biotin-labeled cRNA. The concentration of cRNA was measured by spectrophotometer (Nanodrop, ND-1000, Thermo scientific). Labeled, amplified cRNA (750 ng per array) was hybridized to a ver. 3 of the Illumina Human-Ht-12 BeadChip (48 K) according to the Manufacturer's instructions (Illumina, Inc., San Diego, CA). The whole 48803 probes on the Human-Ht12 beadChip ver. 3 were used and the arrays were scanned with an Illumina Bead array Reader confocal scanner (BeadStation 500GXDW; Illumina, Inc.). Sample Gene Profile option of Illumina BeadStudio software was used to export the gene expression data.

### Wound healing assay

The cells were grown on PLL coated culture plate until confluent in 1% serum medium. A wound was made by scratching a straight line using a 200 µl pipette tip. The cells were then washed twice, transfected and incubated further for 48 hrs with 10% FBS containing media. Images were taken under Phase contrast microscope at 0 hr and 48 hr using AxioObserver fluorescent microscope.

### Matrigel invasion assay

Y79 and Weri-Rb1 cells were counted 24 hr post transfection of the siTiam1 and scramble siRNA and 5×10^4^ cells in serum free media were seeeded respectively in the rehydraded matrigel invasion chamber with 10% FBS containing media added as chemoattractant. Cells were allowed to incubate for 48 hr. Chambers were removed, washed twice with 1× PBS, cleaned the matrigel using cotton plug, fixed cells in methanol, stained with cystal violet. The membranes were cut, removed and mounted with DPX mountant and viewed under 20× objective of Axio-Observer microscope.

### Statistical analysis

Statistical analysis was performed by unpaired students t-test using Graphpad software on the triplicate data generated from three individual experiments. The two-tailed P values less than 0.05 were considered as significant.

## Results

### Differential expression of Tiam1 in RB tumors

In our earlier study, we reported the expression of Tiam1 protein in RB tumors by immunohistochemistry. In this study, we analyzed both the mRNA and protein levels of Tiam1 through qPCR and western blotting. The differential expression of Tiam1 in RB tumors (tumors with choroid invasion (CI) <3 mm and CI>3 mm), Y79 and Weri-Rb1 cell lines were compared to normal cadaveric human retina (n = 2). On average of 7.56±1.8, 3.3±0.3 and 5.3±0.5 fold were expressed higher in RB tumors, Y79 and Weri-Rb1 respectively (Figure 1B) using β-2 microglobulin for normalization in relative quantitative PCR. Tiam1 protein levels were higher in expression compared to the normal retina on Western blot. Tiam1 protein bands were observed at ∼180 KDa, a loading control β-actin was detected at ∼42 KDa in Western blot (Figure 1C). The protein bands intensity were represented as graph in Figure 1D.

### RNA interference of Tiam1 in RB cell lines

**Figure 2:**
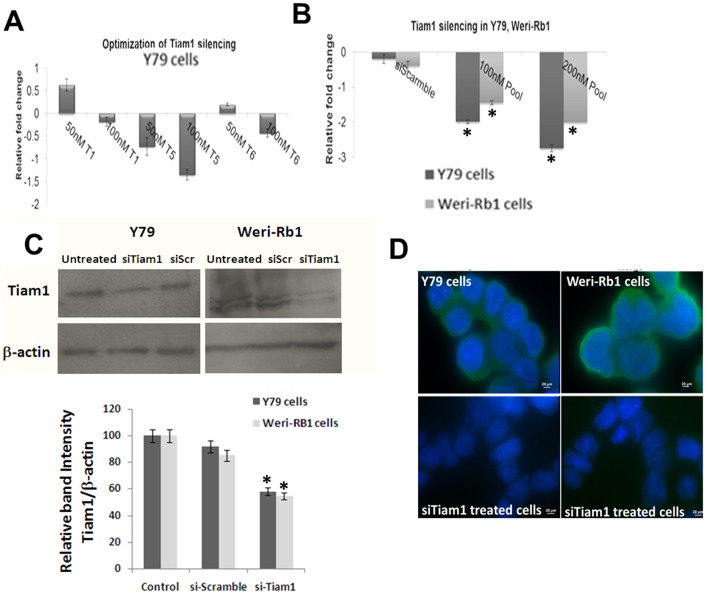
Knockdown of Tiam1 in Y79 and Weri-Rb1 cell lines using RNA interference. **A.** Tiam1 silencing in Y79 cells using three siRNA sequences (T1, T5 & T6) targeting different regions of the mRNA at 50 nM and 100 nM concentrations. **B.** Down-regulation of Tiam1 in Y79 and Weri-Rb1 cells compared to scrambled siRNA mediated by transfection of 100 nM and 200 nM of pooled siRNA. The statistical analysis was calculated using students unpaired t-test and p<0.05 is indicated as asterisk. **C.** Western blot analysis of Tiam1 post silencing in Y79 and Weri-Rb1 cell lines. β-actin showing a band at 42 KDa is used to normalize within sample and between siRNA transfected and untransfected cells, on the right is the densitometry analysis of western blot showing the relative band intensity of Tiam1 expression of siRNA transfected cells compared to scrambled siRNA transfected cells. The statistical analysis was calculated using students unpaired t-test and p<0.05 is indicated as asterisk. **D.** Immunofluorescence images of Tiam1 silenced Y79 and Weri-Rb1 cells showing the reduction of Tiam1 expression on plasma membrane compared to untransfected cells showing high level of Tiam1 expression on plasma membrane. The images were taken at ten fields under 100X oil immersion objective. Scale bar: 20 μm. Tiam1 is shown as green and nucleus counterstained with DAPI fluoresces blue in color.

To study the cellular events mediated by Tiam1 in RB, Tiam1 knockdown is performed transiently in Y79 cells with three different siRNA sequences (T1, T5 and T6) as mentioned in methods. After 48 h of transfection, the silencing efficiency of each siRNA duplexes was determined by Real-time PCR (Figure 2A). Individual siRNA duplexes did not show significant down-regulation of Tiam1 in Y79. As per Parsons et al. (2009) the silencing efficiency was increased when all the three siRNA sequences were pooled and transfected [29]. 200 nM of siRNA pools showed –2.75 and −2.0 fold down regulation in Y79 and Weri-Rb1 respectively (Figure 2B). This was further confirmed at protein level by Western blotting (Figure 2C). The intensity of protein bands were measured using ImageJ software and percentage of Tiam1 expression upon siRNA treatment was calculated and represented as graph. In general, Tiam1 is localized in both plasma membrane and cytoplasm of untransfected cells but the expression level on plasma membrane was drastically reduced upon Tiam1 silencing in Y79 and Weri-Rb1 cells (Figure 2D).

### Tiam1 regulates apoptosis and viability in RB cells

**Figure 3:**
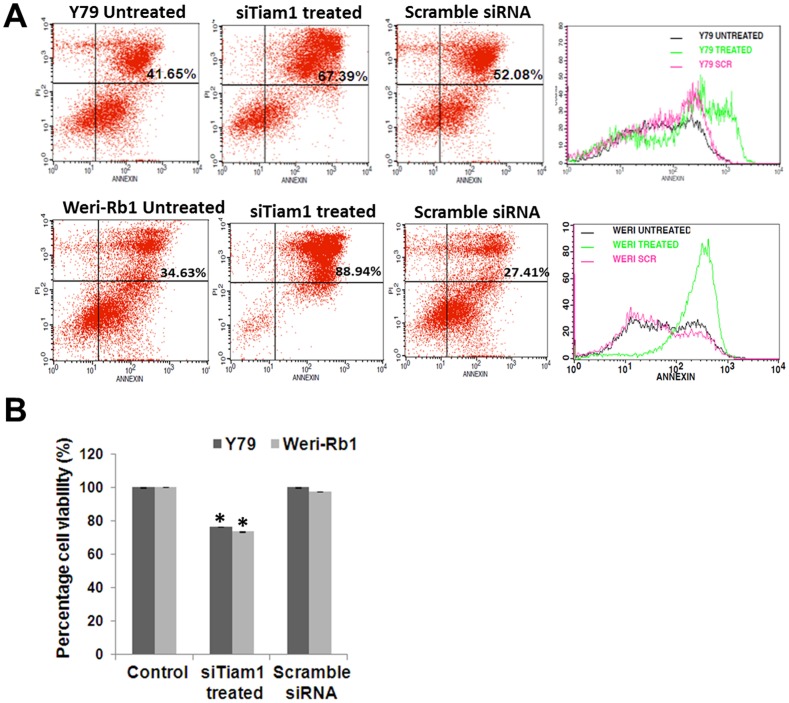
Analysis of Apoptosis and viability of Tiam1 silenced Y79 and Weri-Rb1 cells. **A.** *invitro* cell apoptosis assay showing the difference in mean fluorescence intensity between Tiam1 siRNA treated and Scrambled siRNA treated Y79, Weri-Rb1 cells. On the right panel, overlay graph showing the Annexin V expression between the samples. **B.** MTT assay showing the significant reduction in cell viability in Tiam1 silenced RB cell lines. Error bar represents the standard deviation of triplicates value and the statistical analysis was calculated using students unpaired t-test and p<0.05 is indicated as asterisk.

Further to understand the functional relevance behind the expression of Tiam1 in cellular activities such as apoptosis and viability, studies were carried out in RB cell lines, Y79 and Weri-Rb1 in the presence and absence of Tiam1. To elucidate apoptotic effects upon silencing of Tiam1 Annexin-V assay was performed. Results showed 15% and 50% of late apoptotic cells in Y79 and Weri-Rb1 cells respectively upon siTiam1 (Figure 3A). Upon silencing with Tiam1, the cellular metabolic activity of Y79 and Weri Rb1 decreased by 25% in MTT assay (Figure 3B).

### siRNA mediated Tiam1 silencing results in de-regulation of gene expression in Y79 cells

**Figure 4:**
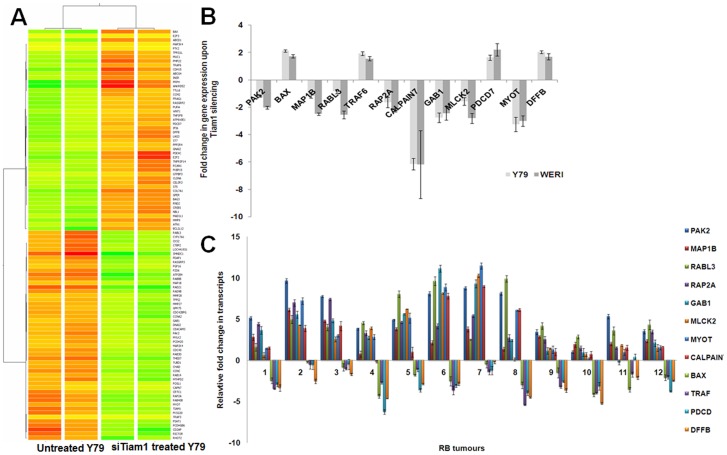
cDNA microarray in Tiam1 silenced Y79 and validation of de-regulated genes in RB cell lines & tumors. **A.** Whole genome microarray of Tiam1 silenced Y79 cells was performed using Human-Ht12 beadChip ver. 3 platform. Hierarchical Cluster represents the expression profile of de-regulated genes upon Tiam1 silencing in Y79 cells, compared to untransfected cells. **B.** Real-time PCR results showing the mRNA expression of selected genes from microarray analysis in Tiam1 silenced Y79 and Weri-Rb1 cells **C.** Relative fold change in the mRNA expression of selected panel of genes verified in primary retinoblastoma showing the negative correlation to that of Tiam1 silenced Y79 and Weri-Rb1 retinoblastoma cells. Error bar represents the standard deviation of triplicates value.

cDNA microarray was performed to analyze the genes regulated upon Tiam1 knock down using Y79 RB cell line. Raw data files were normalized using GeneSpring GX v 12.0. A total of 790 transcripts were observed to be differentially expressed in Tiam1 silenced cells above 1.0 fold (p<0.05) of which 302 genes were up-regulated and 488 genes were down-regulated. Hierarchical clustering of differentially regulated genes was done using Pearson uncentered distance matrix and average linkage rule to establish gene clusters that differentiate the two groups of samples (Figure 4A). Biological analysis of differentially expressed genes was done for Gene Ontology and Pathways using DAVID tool (http://david.abcc.ncifcrf.gov/). Statistically significant ontologies and pathways were filtered based on p-Value <0.05 (Obtained using Fischer Exact Test) with Benjamini Hocheberg FDR correction (Figure S1). The data obtained from microarray analysis was deposited in NCBI's Gene Expression Omnibus (GEO45130) as per MIAME guidelines. The significantly de-regulated genes in response to Tiam1 silencing are listed in Table S1.

### Significantly down-regulated genes in Tiam1 silenced Y79 cells

Since Tiam1 acts as GEF, silencing of Tiam1 led to down- regulation of most of the RAS (rat sarcoma) oncogene family of small GTPases such as RAB8B, RAP2A, RHOT2, RAB3D, RAB14, RAB40B, and RABL3, actin cytoskeleton genes- *Homo sapiens* p21 (CDKN1A)-activated kinase 2 (PAK2), *Homo sapiens* CDC42 binding protein kinase gamma (CDC42BPG), *Homo sapiens* CD2-associated protein (CD2AP), *Homo sapiens* fibroblast growth factor 16 (FGF16), *Homo sapiens* actin-like protein (FKSG30), *Homo sapiens* microtubule-associated protein 1B (MAP1B)., focal adhesion genes- *Homo sapiens* tubulin, delta 1 (TUBD1), Homo sapiens chondroadherin (CHAD), *Homo sapiens* myotilin (MYOT), Homo sapiens myosin light chain kinase 2 (MYLK2), *Homo sapiens* PDGFA associated protein 1 (PDAP1), *Homo sapiens* C-terminal binding protein 2 (CTBP2), *Homo sapiens* CREB regulated transcription coactivator 1 (CRTC1), *Homo sapiens* matrix metallopeptidase 27 (MMP27), *Homo sapiens* matrix metallopeptidase 28 (MMP28), *Homo sapiens* GRB2-associated binding protein 1 (GAB1), *Homo sapiens* calpain 7 (CAPN7).

### Significantly up-regulated genes in Tiam1 silenced Y79 cells

Tiam1 knock down in Y79 cells resulted in up-regulation of apoptotic genes- *Homo sapiens* BCL2-associated X protein (BAX), *Homo sapiens* programmed cell death 7 (PDCD7), *Homo sapiens* DNA fragmentation factor (DFFB), *Homo sapiens* TNF receptor-associated factor 6 (TRAF6), *Homo sapiens* BCL2-like 12 (BCL2L12), GTP binding genes- *Homo sapiens* Rho family GTPase 2 (RND2), *Homo sapiens* RAS guanyl releasing protein 2 (RASGRP2), (multiple drug resistance genes) *Homo sapiens* ATP-binding cassette, sub-family G member 4 (ABCG4), *Homo sapiens* ATP-binding cassette, sub-family D member 1 (ABCD1), *Homo sapiens* cAMP responsive element binding protein 1 (CREB1), and *Homo sapiens* mitogen-activated protein kinase kinase kinase 4 (MAP3K4), Homo sapiens collagen, type VII, alpha 1 (COL7A1), *Homo sapien*s suppression of tumorigenicity 5 (ST5), *Homo sapiens* suppression of tumorigenicity 7 (ST7), *Homo sapiens* tumor protein p63 regulated 1-like (TPRG1L), *Homo sapiens* claudin 6 (CLDN6), *Homo sapiens* cadherin 15 (CDH15).

### Validation of de-regulated genes by qPCR in Tiam1 silenced RB cell lines and primary RB tumors

From the de-regulated genes list, a panel of genes namely PAK2, MAP1B, RABL3, RAP2A, GAB1, MLCK2, MYOT, CALPAIN7, BAX, PDCD, TRAF6, DFFB were selected for the confirmation of microarray analysis by quantitative PCR in Tiam1 silenced Y79 and Weri-Rb1 cells (Figure 4B). The list of primer sequences used for the SYBR-green based qPCR is given in Table 1. Genes involved in actin cytoskeleton were down-regulated where as apoptotic genes were up-regulated in post Tiam1 silenced Y79 and Weri-Rb1 cells. The mRNA expressions of these genes in both RB cell lines were consistent with microarray analysis. Similarly qRT-PCR was done to evaluate the correlation of the validated genes expression in primary RB tumors (Figure 4C). mRNA expression of these selected genes were negatively correlate with primary RB tumors. The average fold expression of 12 genes in 12 tumors normalized to two normal retina were, PAK2 (5.78), MAP1B (2.88), RABL3 (5.00), RAP2A (3.99), GAB1 (4.10), MLCK2 (3.47), MYOT (4.15), CALPAIN7 (3.17), BAX (−2.25), TRAF6 (−2.53), PDCD7 (−2.60), DFFB (−3.00). The list of the primary tumors used and its clinic-pathological descriptions, showing the expression levels of validated genes are given in Table 2. Tumors with CI<3 mm showed lesser extent of changes in gene expression compared to tumors with CI>3 mm (tumor 2, 3, 5, 6, 7). qPCR results were shown in Figure 4 & CI status were shown in Table 2. The expression levels of Tiam1 is also directly correlating to the changes in gene expression of validated targets in RB tumor.

**Table 1:** Primer sequences for the selected panel of validated genes.

GENE	PRIMER SEQUENCES	
RAP2A	FP: AGA TCA TCC GCG TGA AGC	RP: CCC CAC TCT TCA GCA AGG
BAX	FP: TGG AGCTGCAGAGGATGATTG	RP: GAAGTTGCCGTCAGAAAACATG
Rabl3	FP: TTGGGAGACTCAGGTGTTGGGAAA	RP: CAGTTGGCACCAAATCCCTGTTGA
PAK2	FP: GAATGGAAGGATCTGTTAAGCTCACT	RP: GCCATAAGCTTTCCGTGTAACC
MAP1B	FP: AAAGTGTCCAGGGTGGCTTC	RP: CTCCTGGTACCATTCCCTCA
TRAF6	FP: TGGCATTACGAGAAGCAGTG	RP: GTTCCATCTTGTGCAAACAACC
DFFB	FP: GGCCTGCTTTTTACCTCAGA	RP: CGTTTCCGCACAGGCTGCTT
MLCK2	FP: GAGCTGAGGACCGGGAAT	RP: AGGTACAGACTGCCCCAAAC
CALPAIN7	FP: ATCTGGAAAGAGTTCAAGCT	RP: GCACGCTCTAAGACCAACAG
PDCD7	FP: TTGACCCAGGCTGCCTAT	RP: AATCTCCTGCTCGCGTTC
MYOT	FP: CGACTGCAAGTTCCTACATCAC	RP: TGAATGAAACGTGGTGGGTA
GAB1	FP: ACCTCAAGCCAGACAGAAAAGT	RP: TCGAGCAAAACTCCTAGTGATG

**Table 2:** Clinicopathological features of primary Retinoblastoma tumors and gene expression profile by QRT-PCR.

S.No.	Age/ Sex	Clinicopathological descriptions	PAK2	MAP1B	RABL3	RAP2A	GAB1	MLCK2	MYOT	CALPAIN7	BAX	TRAF6	PDCD7	DFFB
1	2/M	RB, PD, Focal CI<3mm, Pre laminar& Post laminar invasion of ON.	UR	UR	UR	UR	UR	NS	UR	UR	DN	DN	DN	DN
2	2/M	RB, UD, CI>3mm, Tumor cells invading in anterior border of sclera.	UR	UR	UR	UR	UR	UR	UR	UR	NS	NS	NS	DN
3	2/F	RB, PD, CI>3mm, Pre laminar & Post laminar invasion of tumor.	UR	UR	UR	UR	UR	UR	UR	UR	NS	DN	NS	DN
4	2/F	RB, WD, CI<3mm, No invasion of ON.	UR	NS	UR	UR	UR	UR	UR	NS	DN	DN	DN	DN
5	6/M	RB, CI>3mm, Tumor cells touching the anterior border of the sclera.	UR	UR	UR	UR	UR	UR	UR	UR	DN	DN	DN	DN
6	3/F	RB, MD, CI>3mm, Pre laminar& Post laminar invasion of ON.	UR	UR	UR	UR	UR	UR	UR	UR	DN	DN	DN	DN
7	4/M	RB, PD, CI>3mm, Pre laminar& Post laminar invasion, Tumor invasion into anterior, middle& posterior border of sclera.	UR	UR	UR	UR	UR	UR	UR	UR	NS	DN	DN	NS
8	1/F	RB, WD, CI<3mm, No invasion of ON	UR	UR	UR	UR	UR	NS	UR	UR	DN	DN	DN	DN
9	3/F	RB, PD, No CI, Pre laminar &Post laminar invasion of ON.	UR	UR	UR	UR	UR	UR	UR	UR	DN	DN	DN	DN
10	2/M	RB, PD, NoCI, Pre laminar invasion of ON.	UR	UR	UR	UR	NS	NS	NS	NS	DN	DN	DN	DN
11	13mon/ M	RB, WD, NoCI, No invasion of ON	UR	UR	UR	UR	NS	UR	NS	UR	DN	DN	NS	DN
12	11/F	RB, MD, CI<3mm	UR	UR	UR	UR	UR	UR	UR	UR	DN	DN	DN	DN

F: Female; M: Male; RB: Retinoblastoma; PD: Poorly Differentiated; MD: Moderately Differentiated; WD: Well Differentiated; UD: Un-Differentiated; CI: Choroid Invasion; ON: Optic Nerve; UR: Up-regulation above 1 fold change (Log 2 ratio); DR: Down-regulation above 1 fold change (Log2 ratio); NS: Not Significant fold change.

### Tiam1 regulates actin polymerization, cell migration and invasion in RB cell lines

**Figure 5:**
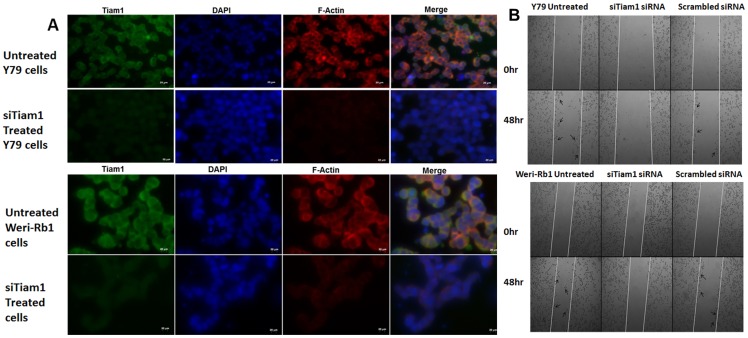
F-actin staining and invasion of Tiam1 deficient RB cell lines. **A.** Tiam1 silenced Y79 cells and Weri-Rb1 cells were fixed, immunofluorescently labeled for Tiam1, nucleus stained with DAPI, stained with phalloidin and images were taken at 40X in ten fields. Bar represents 20 μm. **B.** Phase contrast microscope images of wound healing assay showing the cell migration pattern in Tiam1 deficient retinoblastoma cell lines at 0 hr and 48 hrs post silencing. Tiam1 knockdown cells were unable to migrate whereas the untransfected and control cells showed increased migration, migrated cells were indicated with arrows. The images were acquired using AxioObserver microscope at 5× objective with 1× optovar.

**Figure 6:**
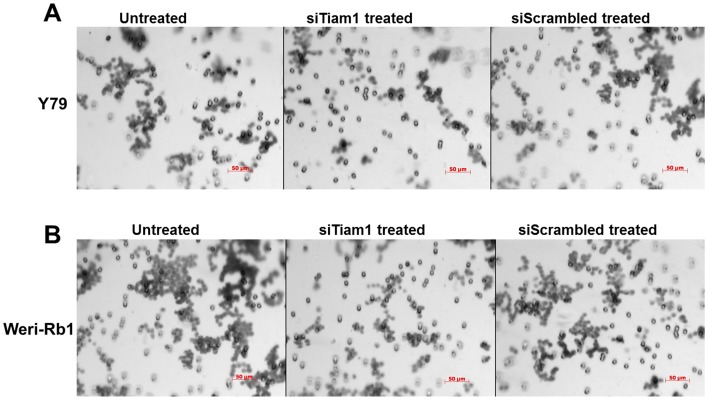
Matrigel invasion assay of Tiam1 deficient RB cell lines. **A**. Y79 panel showing the invasion of untransfected, siTiam1 treated and scrambled siRNA treated cells. **B.** Weri-Rb1 cells. The matrigel invasion chambers post invasion was stained with crystal violet and images acquired at 20× objective. The experimental results were representative of triplicate result repeated twice.

To investigate the involvement of Tiam1 in actin cytoskeleton regulation, F-actin staining was performed using phalloidin in Tiam1 silenced Y79 and Weri-Rb1 cells (Figure 5A). As mentioned above, Tiam1 was co-localized with actin at cell junctions. In case of Tiam1 knockdown, the cells exhibited lesser extent of actin polymerization at the cellular junctions and actin co-localization. Thus the results show that Tiam1 is essential for actin re-organisation in RB cells. Additionally, the function of Tiam1 in cell migration was assessed by wound healing assay (Figure 5B). Tiam1 silenced RB cell lines showed impairment of cell migration, compared to untransfected and scrambled siRNA transfected RB cells. Silencing of Tiam1 in Weri-Rb1 had resulted in lesser number of cells invaded across the matrigel coated invasion chamber. This indicated the decrease in invasion potential depends on Tiam1 expression as it is not observed in the scramble siRNA transfected cells (Figure 6).

### Plasma membrane localization of Tiam1 is mediated by N-terminal PH domain

**Figure 7:**
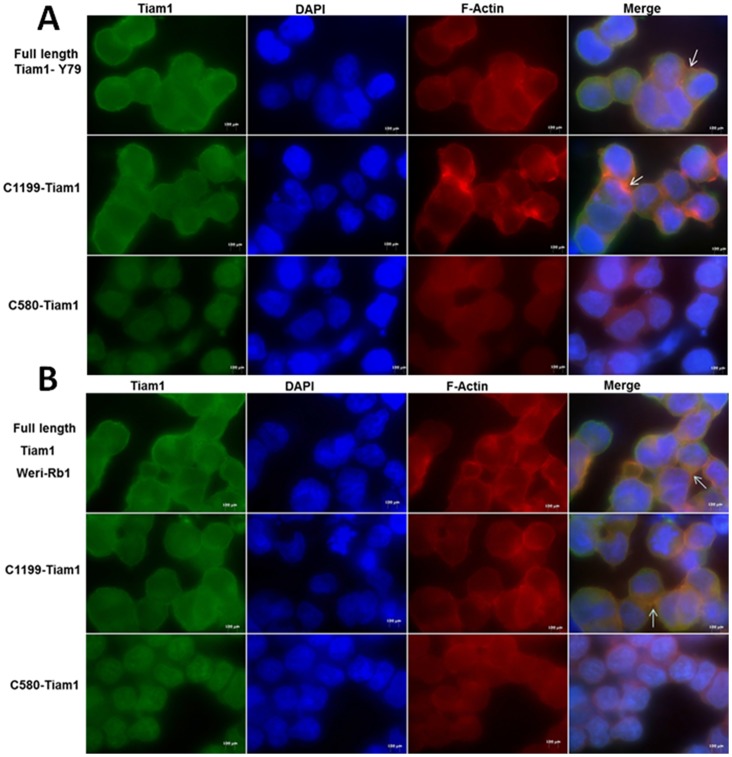
Localization of Full length Tiam1, C1199 Tiam1 and C580 Tiam1 in RB cell lines. A) Immunofluorescent images of Y79 cells, B) Weri-Rb1 cells transfected with Full length Tiam1, C1199 Tiam1 and C580 Tiam1 plasmids tagged with HA. Tiam1 and F-actin were stained as mentioned in the materials and methods. Full length Tiam1 and C1199 Tiam1 but not C580 Tiam1 were localized to plasma membrane. Unlike Full length Tiam1 and C1199 Tiam1, C580 Tiam1 could not induce membrane ruffling. Representative images were taken from 10 independent fields using AxioObserver fluorescent microscope at 100× and arrows indicate the membrane ruffling. Scale bar: 20 μm.

Since Tiam1 localizes along with F-actin and controls the actin cytoskeleton, we investigated which domain of the protein regulates the localization Tiam1 on plasma membrane in RB. RB cell lines (Y79 and Weri-Rb1) transfected with full length and C1199 Tiam1 showed the membrane localization and induced the membrane ruffling (Figure 7). In contrast, C580 Tiam1 was localized to the nucleus and failed to induce membrane ruffling.

### N-terminal PH domain regulates retinoblastoma cell motility

**Figure 8:**
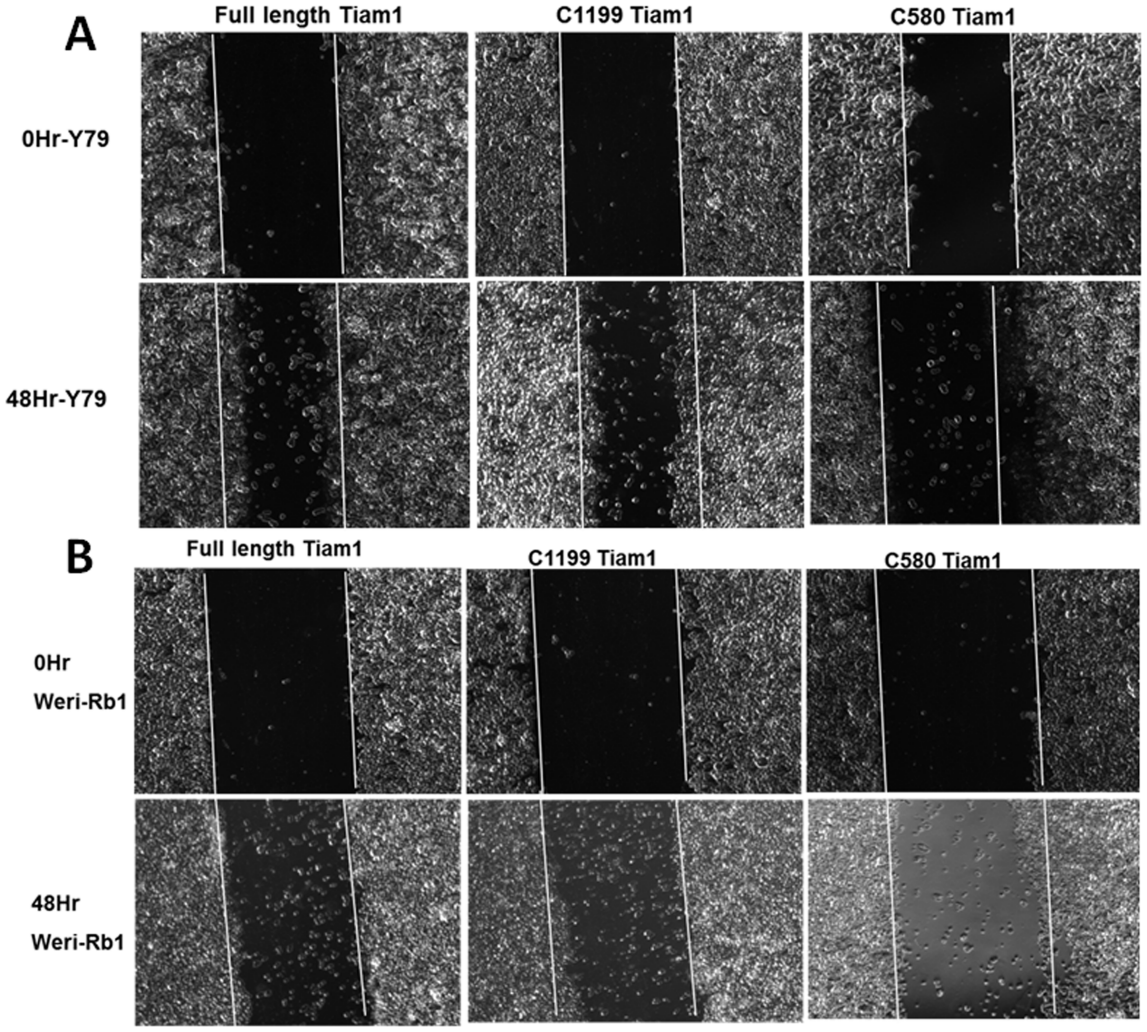
N-terminal PH domain maintains cell motility in retinoblastoma cells. Phase contrast images showing the migration of Y79 and Weri-Rb1 cells transfected with Full length Tiam1, C1199 Tiam1 and C580 Tiam1 in wound-healing assay. White line indicates the original wound edge which made by a pipette tip. The images were taken from 10 different locations. Cells transfected with full length and C1199 Tiam1 showing significant increase in cell migration rate compared to the cells transfected with C580 Tiam1. The Images were captured at 5× objective in AxioObserver microscope.

We observed that N-terminal PH domain, but not C-terminal PH domain modulates the localization of Tiam1 and induces membrane ruffling in RB cells. Further we determined the association of membrane localization of Tiam1 and cell migration in Y79, Weri-Rb1 cells. To elucidate this, wound healing assay was performed in Y79 and Weri-Rb1 cells. The cells transfected with full length Tiam1 and C1199 Tiam1 were showing more cell migration towards the wound, whereas C580 Tiam1 transfected cells showed delayed cell migration into the wound in both cell lines (Figure 8).

## Discussion

**Figure 9:**
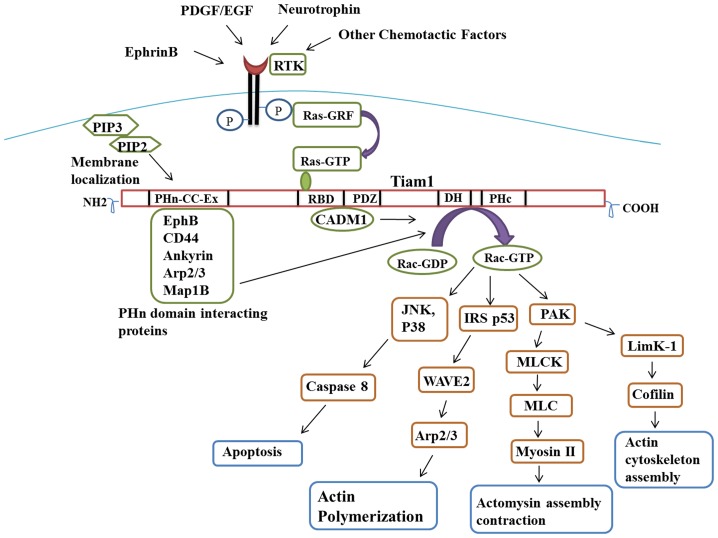
Tiam1 mediated signaling pathway. Schematic representation showing the upstream and downstream effects of Tiam1 in intracellular pathways. External stimulants phosphorylate the receptor tyrosine kinase which inturn activates Ras-GTP, binds to RBD domain of Tiam1. Interaction of EphB, CD44 and Ankyrin Proteins with PHn domain of Tiam1 and CADM1 with PDZ domain leads to the activation of Rac-GTP at DH domain of Tiam1. The activated Rac-GTP involves in various signaling pathways, mainly on actin cytoskeleton. The binding of PHn domain to phosphoinositide facilitates the membrane localization of Tiam1. PDGF: Platelet-derived growth factor; EGF: Epidermal growth factor; RTK: Receptor tyrosine kinase; PIP3: Phosphatidyl inositol triphosphate; PIP2: Phosphatidyl inositol diphosphate; PHn: N-terminal PH domain; RBD: Ras binding domain; PDZ: PSD-95/DglA/ZO-1 domain; DH: Dgl homology domain; PHc: C-terminal PH domain; JNK: Jun N-terminal kinase; PAK: p21-activated kinase.

Though Tiam1 has been shown to play a crucial role in actin cytoskeleton, cell migration and invasion, various intracellular pathways are involved in activation of upstream and downstream signaling of Tiam1 (Figure 9) [9], [10], [13], [27]. In particular, Tiam1 is required for activation of Rac1 and cdc42 to produce specific actin rich structures like membrane ruffling, lamellipodia, filopodia [30], [31], [32], [33], [34], [35] and for neurite outgrowth [36]. Addition to that, Tiam1 interacts with CADM1, Ephrin and Arp2/3 to initiate Rac1 mediated actin cytoskeleton remodeling [37], [38], [39]. Hence in the current study, we analysed the importance of Tiam1 in RB cells using short interfering RNA (siRNA) mediated knockdown studies.

Silencing of Tiam1 in RB cell lines followed by cDNA microarray showed various pathways and genes altered, predominantly genes related to MAPK pathway, small GTPase, apoptosis and cell migration. The impairment of cell migration in Tiam1 silenced Y79 and Weri-Rb1 cells was proved by Wound healing and matrigel invasion assay. This correlates with the microarray analysis where the actin cytoskeleton genes were down-regulated in Tiam1 silenced Y79 cells resulting in lesser cellular migration potential (Figure 5). One of the down-regulated actin cytoskeleton gene in our microarray analysis is MAP1B (Microtubule-associated protein 1B) which is needed for axonal development, reported to be involved in neurite growth, neuron migration and metastasis [40], [41], [42]. MAP1B is found to interact with Tiam1 thereby activating Rac1 and cdc42 and further inhibiting RhoA activity which leads to actin polymerization and axonal elongation [43], [44]. MAP1B deficient cells exhibit a decreased cell migration and axonal development [45], [46]. The other mechanism might be PAK mediated activation of MyosinII, a protein involved in stress fiber formation and contraction [47]. The phosphorylation of myosinII light chain (MLC) by myosinII light chain kinase (MLCK) regulates actin-myosin II interaction [48]. We observed that PAK2 and MLCK were down-regulated in Tiam1 silenced retinoblastoma Y79 cells. Moreover, small GTPase subfamily Rab-like 3 (Rabl3) and actin-binding protein Myotilin, promote motility, tumor cell survival [49]. The down-regulation of Rabl3 and myotilin might as well attribute to the suppression of cell motility in Tiam1 silenced cells. The expression level of these genes when validated in primary RB tumors showed differential expression correlating with their CI status. CI represents the invasion potential of the given tumor during the enucleation, which may or may not have undergone chemotherapeutic treatment. The tumors with CI>3 mm compared to CI<3 mm cases showed increased expression of pro-survival genes and decreased expression of apoptotic genes.

From our Annexin-V assay results, a remarkable increase in apoptosis was observed. The mechanism of apoptotic induction might be due the up-regulation of pro-apoptotic gene BAX (Bcl-2 associated X), PDCD7 (Programmed cell death protein), TRAF6 (Tumor necrosis factor receptor-associated factor 6) and DFFB (DNA fragmentation factor subunit beta) upon Tiam1 knockdown [50], [51], [52], [53], [54]. Similar to our results, apoptotic cell death has been observed earlier upon Tiam1 silencing [55].

Since endogenous Tiam1 is localized in both plasma membrane and cytoplasm, we were interested to find out which domain of the Tiam1 protein regulates the localization intracellularly in RB cells. We elucidated that N-terminal PH domain of Tiam1 mediates the membrane localization and invasion but not the C-terminal PH domain and targeting the N-terminal PH domain of Tiam1 affects the cell migration and invasion. Earlier studies showed that localization of Tiam1 to plasma membrane requires N-terminal PH domain. Also the membrane localization of Tiam1 is required for the activation of the c-Jun NH_2_-terminal kinase (JNK) thus leading to successful Rac mediated signaling [13]. N-terminal PH domain of Tiam1 has affinity towards phosphoinositides and its interaction with transmembrane domain accelerates the membrane localization. For the first time our data has additionally elucidated the importance of the N-terminal region with respect to the migration as it is directly correlating with actin cytoskeleton modulation in RB. From our findings, we suggest that Tiam1 can be utilized as a potential target for RB therapy. In future, docking studies to find potent inhibitors to RBD binding or alternatively aptamers targeting the N-terminal PH domain of Tiam1 need to be isolated, which have better application *in vivo* for drug delivery purposes.

## Supporting Information

Figure S1 **Pathways and gene ontology altered by Tiam1.** A. Pie chart represents significantly de-regulated pathways in response to Tiam1 silencing. B. Graphical representation of significantly de-regulated gene ontology.
(TIF)

Table S1 **Deregulated genes in Tiam1 silenced Y79 cells.** List of genes shortlisted and used for Hierarchial clustering and further pathway and gene ontology analysis.
(DOC)
