# Expression of Concern: RNAi Mediated Tiam1 Gene Knockdown Inhibits Invasion of Retinoblastoma

**DOI:** 10.1371/journal.pone.0252495

**Published:** 2021-05-26

**Authors:** The PLOS ONE Editors

Following the publication of this article [1], concerns were raised regarding results presented in Figs 5, 6 and 8. Specifically,

**Fig 5:**
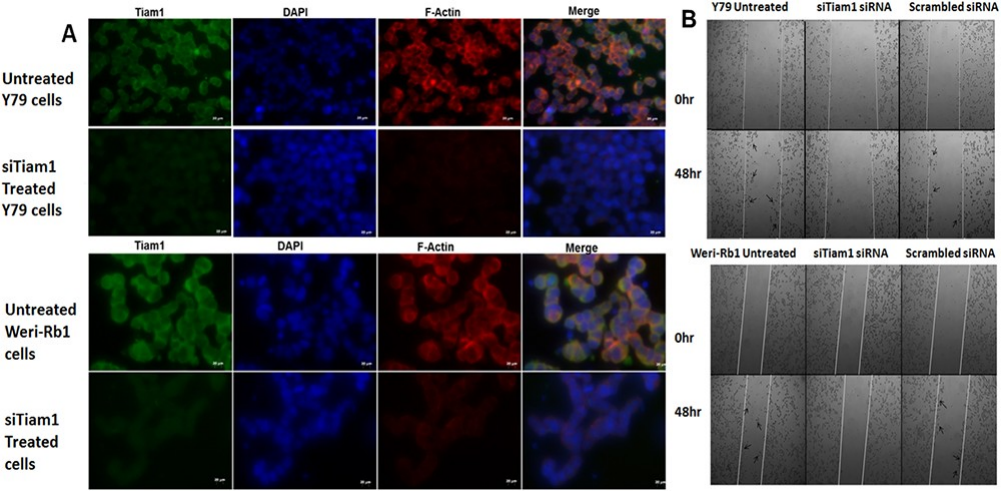
F-actin staining and invasion of Tiam1 deficient RB cell lines. **A**. Tiam1 silenced Y79 cells and Weri-Rb1 cells were fixed, immunofluorescently labeled for Tiam1, nucleus stained with DAPI, stained with phalloidin and images were taken at 40X in ten fields. Bar represents 20 μm. **B**. Phase contrast microscope images of wound healing assay showing the cell migration pattern in Tiam1 deficient retinoblastoma cell lines at 0 hr and 48 hrs post silencing. Tiam1 knockdown cells were unable to migrate whereas the untransfected and control cells showed increased migration, migrated cells were indicated with arrows. The images were acquired using AxioObserver microscope at 5× objective with 1× optovar.

**Fig 6:**
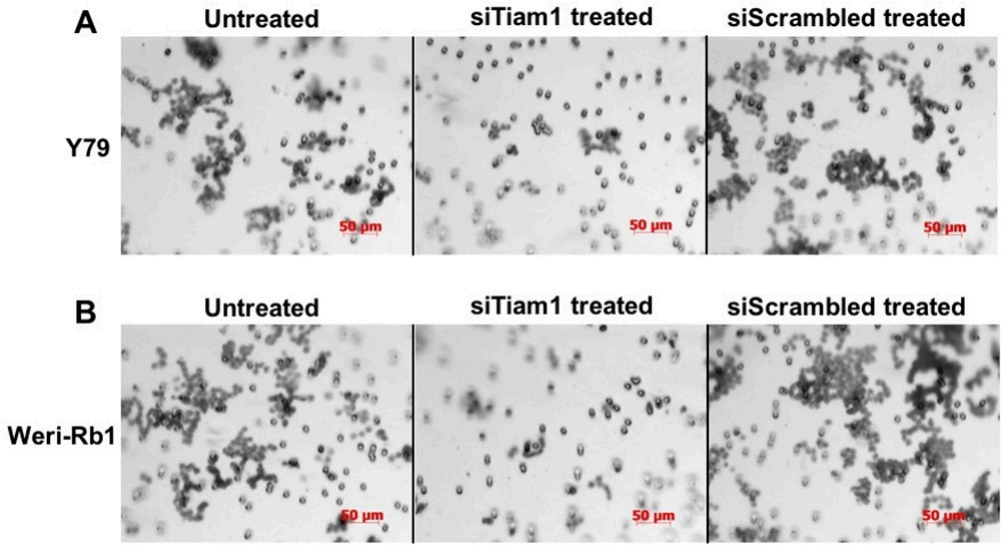
Matrigel invasion assay of Tiam1 deficient RB cell lines. **A**. Y79 panel showing the invasion of untransfected, siTiam1 treated and scrambled siRNA treated cells. **B**. Weri-Rb1 cells. The matrigel invasion chambers post invasion was stained with crystal violet and images acquired at 20× objective. The experimental results were representative of triplicate result repeated twice.

**Fig 8:**
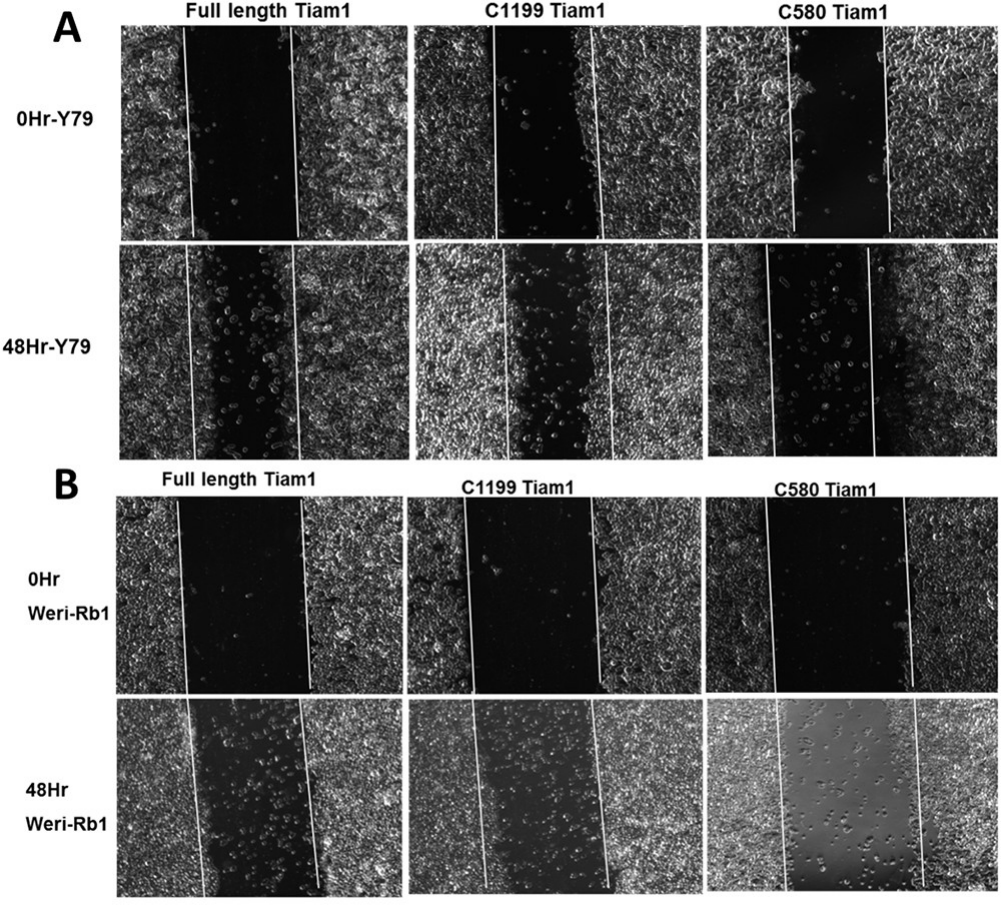
N-terminal PH domain maintains cell motility in retinoblastoma cells. Phase contrast images showing the migration of Y79 and Weri-Rb1 cells transfected with Full length Tiam1, C1199 Tiam1 and C580 Tiam1 in wound-healing assay. White line indicates the original wound edge which made by a pipette tip. The images were taken from 10 different locations. Cells transfected with full length and C1199 Tiam1 showing significant increase in cell migration rate compared to the cells transfected with C580 Tiam1. The Images were captured at 5× objective in AxioObserver microscope.

- In Fig 5B, the 0hr Y79 Untreated panel and the 0hr Scrambled siRNA panel appear similar.
- The Y79 siTiam 1 treated panel of Fig 6A partially overlaps with the Weri-Rb1 siScrambled treated panel of Fig 6B.
- The Y79 siScrambled treated panel of Fig 6A partially overlaps with the Weri-Rb1 untreated and siTiam 1 treated panels of Fig 6B.
- The 0Hr-Y79 C1199 Tiam 1 panel of Fig 8A appears similar to the 0Hr-Weri-Rb1 Full length Tiam 1 panel of Fig 8B.

The corresponding authors indicated that errors were made during the compilations of Figs 5 and 8 resulting in accidental duplications of the Fig 5B 0hr Y79 Untreated panel and the Fig 8B 0hr Weri-Rb1 Full length Tiam1 panel. Revised Figs 5 and 8 provided with this notice include updates to the Fig 5B 0hr Scrambled siRNA panel and the Fig 8A 0Hr-Y79 C1199 Tiam1 panel showing results from the original experiments.

In addition, the corresponding authors explained that the partial overlap of panels presented in Fig 6 was the result of mixing files with similar names. A revised Fig 6 is provided to update the Y79 siTiam 1 treated, siScrambled treated, and Weri-Rb 1 siTiam1 treated panels showing results from the original experiment.

The raw data underlying all other results reported in the article are available upon request from the corresponding authors.

Updated Figs 5, 6 and 8 and their respective figure captions are provided here. A member of *PLOS ONE*’s Editorial Board advised that the updated figures support the results and conclusions reported in the original article. However, the *PLOS ONE* Editors issue this Expression of Concern due to the number of panels and figures affected and concerns regarding the overall reliability of the data presented in the original article.
