# Supplementary figures and images for: RNAi Mediated Tiam1 Gene Knockdown Inhibits Invasion of Retinoblastoma
Source: PLoS One. 2013 Aug 7;8(8):e70422. doi: 10.1371/journal.pone.0070422 (PMC3737373; doi:10.1371/journal.pone.0070422)

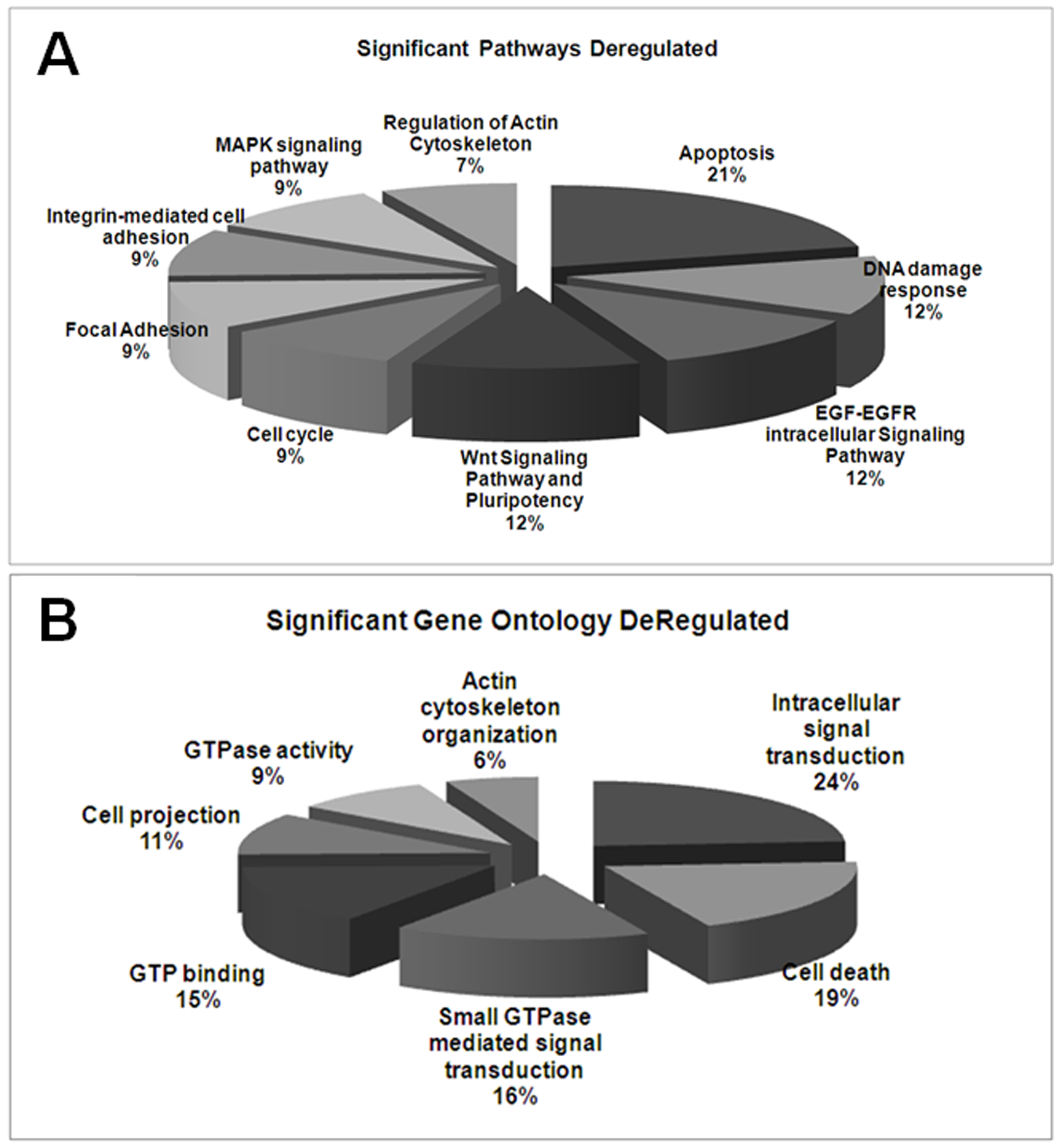

Supplement: Figure S1 — Pathways and gene ontology altered by Tiam1. A. Pie chart represents significantly de-regulated pathways in response to Tiam1 silencing. B. Graphical representation of significantly de-regulated gene ontology. (TIF) [file pone.0070422.s001.tif]
