# Supplementary material for: RNAi Mediated Tiam1 Gene Knockdown Inhibits Invasion of Retinoblastoma
Source: PLoS One. 2013 Aug 7;8(8):e70422. doi: 10.1371/journal.pone.0070422 (PMC3737373; doi:10.1371/journal.pone.0070422)
Supplement: Table S1 — Deregulated genes in Tiam1 silenced Y79 cells. List of genes shortlisted and used for Hierarchial clustering and further pathway and gene ontology analysis. (DOC) [file pone.0070422.s002.doc]

**Table S**1. Deregulated genes in Tiam1 silenced Y79 cells.

| **GeneID** | **Symbol** | **Gene Name** | **Chromosome location** | **Fold change** | **Regulation** |
| --- | --- | --- | --- | --- | --- |
| **Apoptosis** | | | | | |
| **581** | BAX | BCL2-associated X protein | 19q13.3-q13.4 | 1.17 | up |
| **5062** | PAK2 | p21 protein (Cdc42/Rac)-activated kinase 2 | 3q29 | 1.13 | down |
| **5747** | PTK2 | PTK2 protein tyrosine kinase 2 | 8q24.3 | 1.03 | up |
| **1677** | DFFB | DNA fragmentation factor, 40kDa, beta polypeptide (caspase-activated DNase) | 1p36.3 | 1.14 | up |
| **6416** | MAP2K4 | Mitogen-activated protein kinase kinase 4 | 17p12 | 1.08 | down |
| **7189** | TRAF6 | TNF receptor-associated factor 6, E3 ubiquitin protein ligase | 11p12 | 1.10 | up |
| **4216** | MAP3K4 | Mitogen-activated protein kinase kinasekinase 4 | 6q26 | 1.04 | up |
| **83596** | BCL2L12 | BCL2-like 12 (proline rich) | 19q13.3 | 1.17 | up |
| **9531** | BAG3 | BCL2-associated athanogene 3 | 10q25.2-q26.2 | 1.14 | up |
| **7187** | TRAF3 | TNF receptor-associated factor 3 | 14q32.32 | 1.03 | down |
| **Intracellular signal transduction** | | | | | |
| **1385** | CREB1 | cAMP responsive element binding protein 1 | 2q34 | 1.15 | up |
| **7074** | TIAM1 | T-cell lymphoma invasion and metastasis 1 | 21q22.11 | 1.16 | down |
| **1586** | CYP17A1 | Cytochrome P450, family 17, subfamily A, polypeptide 1 | 10q24.3 | 1.16 | down |
| **1164** | CKS2 | CDC28 protein kinase regulatory subunit 2 | 9q22 | 1.14 | down |
| **83605** | CCM2 | Cerebral cavernous malformation 2 | 7p13 | 1.12 | up |
| **3643** | INSR | Insulin receptor | 19p13.3-p13.2 | 1.11 | up |
| **283373** | ANKRD52 | Ankyrin repeat domain 52 | 12q13.3 | 1.29 | up |
| **493** | ATP2B4 | ATPase, Ca++ transporting, plasma membrane 4 | 1q32.1 | 1.24 | down |
| **11333** | PDAP1 | PDGFA associated protein 1 | 7q22.1 | 1.16 | down |
| **215** | ABCD1 | ATP-binding cassette, sub-family D (ALD), member 1 | Xq28 | 1.14 | up |
| **23373** | CRTC1 | CREB regulated transcription coactivator 1 | 19p13.11 | 1.13 | down |
| **1827** | RCAN1 | Regulator of calcineurin 1 | 21q22.12 | 1.12 | up |
| **944** | TNFSF8 | Tumor necrosis factor (ligand) superfamily, member 8 | 9q33 | 1.11 | up |
| **7980** | TFPI2 | Tissue factor pathway inhibitor 2 | 7q22 | 1.10 | down |
| **8823** | FGF16 | Fibroblast growth factor 16 | Xq13 | 1.09 | down |
| **2549** | GAB1 | GRB2-associated binding protein 1 | 4q31.21 | 1.08 | down |
| **Adhesion molecule** | | | | | |
| **1294** | COL7A1 | Collagen, type VII, alpha 1 | 3p21.1 | 1.19 | up |
| **1101** | CHAD | Chondroadherin | 17q21.33 | 1.12 | down |
| **85366** | MYLK2 | Myosin light chain kinase 2 | 20q13.31 | 1.10 | down |
| **1013** | CDH15 | Cadherin 15, type 1, M-cadherin (myotubule) | 16q24.3 | 1.20 | up |
| **56100** | PCDHGB6 | Protocadherin gamma subfamily B, 6 | 5q31 | 1.16 | down |
| **1951** | CELSR3 | Cadherin, EGF LAG seven-pass G-type receptor 3 (flamingo homolog, Drosophila) | 3p21.31 | 1.13 | up |
| **4582** | MUC1 | Mucin 1, cell surface associated | 1q21 | 1.12 | up |
| **1084** | CEACAM3 | Carcinoembryonic antigen-related cell adhesion molecule 3 | 19q13.2 | 1.11 | down |
| **9074** | CLDN6 | Claudin 6 | 16p13.3 | 1.11 | up |
| **64881** | PCDH20 | Protocadherin 20 | 13q21 | 1.10 | down |
| **23473** | CAPN7 | Calpain 7 | 3p24 | 1.06 | down |
| **Cell death** | | | | | |
| **2537** | IFI6 | Interferon, alpha-inducible protein 6 | 1p35 | 1.11 | up |
| **10285** | SMNDC1 | Survival motor neuron domain containing 1 | 10q23 | 1.34 | down |
| **5885** | RAD21 | RAD21 homolog (S. pombe) | 8q24 | 1.27 | down |
| **8764** | TNFRSF14 | Tumor necrosis factor receptor superfamily, member 14 | 1p36.32 | 1.13 | up |
| **10081** | PDCD7 | Programmed cell death 7 | 15q22.31 | 1.12 | up |
| **1822** | ATN1 | Atrophin 1 | 12p13.31 | 1.10 | up |
| **GPCR ligand binding** | | | | | |
| **2771** | GNAI2 | Guanine nucleotide binding protein (G protein), alpha inhibiting activity polypeptide 2 | 3p21.31 | 1.09 | up |
| **2852** | GPER | G protein-coupled estrogen receptor 1p | 7p22.3 | 1.15 | up |
| **10936** | GPR75 | G protein-coupled receptor 75 | 2p16 | 1.12 | down |
| **5143** | PDE4C | Phosphodiesterase 4C, cAMP-specific | 19p13.11 | 1.19 | up |
| **Small GTPase mediated signal transduction** | | | | | |
| **51762** | RAB8B | RAB8B, member RAS oncogene family | 15q22.2 | 1.18 | down |
| **5911** | RAP2A | RAP2A, member of RAS oncogene family | 13q34 | 1.17 | down |
| **89941** | RHOT2 | Ras homolog family member T2 | 16p13.3 | 1.16 | down |
| **9545** | RAB3D | RAB3D, member RAS oncogene family | 19p13.2 | 1.13 | down |
| **8153** | RND2 | Rho family GTPase 2 | 17q21 | 1.12 | up |
| **51552** | RAB14 | RAB14, member RAS oncogene family | 9q32-q34.11 | 1.12 | down |
| **10966** | RAB40B | RAB40B, member RAS oncogene family | 17q25.3 | 1.17 | down |
| **285282** | RABL3 | RAB, member of RAS oncogene family-like 3 | 3q13.33 | 1.14 | down |
| **25780** | RASGRP3 | RAS guanyl releasing protein 3 (calcium and DAG-regulated) | 2p25.1-p24.1 | 1.12 | down |
| **10235** | RASGRP2 | RAS guanyl releasing protein 2 (calcium and DAG-regulated) | 11q13 | 1.11 | up |
| **51174** | TUBD1 | Tubulin, delta 1 | 17q23.1 | 1.11 | down |
| **144715** | RAD9B | RAD9 homolog B (S. pombe) | 12q24.11 | 1.15 | down |
| **Actin cytoskeleton organization** | | | | | |
| **55561** | CDC42BPG | CDC42 binding protein kinase gamma (DMPK-like) | 11q13.1 | 1.14 | down |
| **23607** | CD2AP | CD2-associated protein | 6p12 | 1.23 | down |
| **253260** | RICTOR | RPTOR independent companion of MTOR, complex 2 | 5p13.1 | 1.17 | down |
| **23307** | FKBP15 | FK506 binding protein 15, 133kDa | 9q32 | 1.13 | up |
| **5630** | PRPH | Peripherin | 12q12-q13 | 1.26 | up |
| **5376** | PMP22 | Peripheral myelin protein 22 | 17p12 | 1.17 | up |
| **440915** | FKSG30 | POTE ankyrin domain family, member K, pseudogene | 2q21.1 | 1.13 | down |
| **284076** | TTLL6 | Tubulin tyrosine ligase-like family, member 6 | 17q21.32 | 1.11 | up |
| **9499** | MYOT | Myotilin | 5q31 | 1.10 | down |
| **4131** | MAP1B | Microtubule-associated protein 1B | 5q13 | 1.08 | down |
| **Metallopeptidase** | | | | | |
| **4318** | MMP9 | Matrix metallopeptidase 9 (gelatinase B, 92kDa gelatinase, 92kDa type IV collagenase) | 20q11.2-q13.1 | 1.17 | up |
| **64066** | MMP27 | Matrix metallopeptidase 27 | 11q24 | 1.13 | down |
| **79148** | MMP28 | Matrix metallopeptidase 28 | 17q21.1 | 1.08 | down |
| **Wnt signaling** | | | | | |
| **8061** | FOSL1 | FOS-like antigen 1 | 11q13 | 1.08 | down |
| **5524** | PPP2R4 | Protein phosphatase 2A activator, regulatory subunit 4 | 9q34 | 1.07 | up |
| **7471** | WNT1 | Wingless-type MMTV integration site family, member 1 | 12q13 | 1.07 | up |
| **8323** | FZD6 | Frizzled family receptor 6 | 8q22.3-q23.1 | 1.16 | down |
| **1488** | CTBP2 | C-terminal binding protein 2 | 10q26.13 | 1.14 | down |
| **8323** | FZD6 | Frizzled family receptor 6 | 8q22.3-q23.1 | 1.16 | down |
| **1488** | CTBP2 | C-terminal binding protein 2 | 10q26.13 | 1.14 | down |
| **Cell cycle** | | | | | |
| **1870** | E2F2 | E2F transcription factor 2 | 1p36 | 1.21 | up |
| **1871** | E2F3 | E2F transcription factor 3 | 6p22 | 1.02 | up |
| **890** | CCNA2 | Cyclin A2 | 4q27 | 1.14 | down |
| **892** | CCNC | Cyclin C | 6q21 | 1.18 | down |
| **51362** | CDC40 | Cell division cycle 40 | 6q21 | 1.13 | down |
| **8379** | MAD1L1 | MAD1 mitotic arrest deficient-like 1 (yeast) | 7p22 | 1.08 | up |
| **Lymphocyte tarbase** | | | | | |
| **51014** | TMED7 | Transmembrane emp24 protein transport domain containing 7 | 5q22.3 | 1.20 | down |
| **10797** | MTHFD2 | Methylenetetrahydrofolate dehydrogenase (NADP+ dependent) 2, methenyltetrahydrofolatecyclohydrolase | 2p13.1 | 1.16 | down |
| **29968** | PSAT1 | Phosphoserine aminotransferase 1 | 9q21.2 | 1.14 | down |
| **8992** | ATP6V0E1 | ATPase, H+ transporting, lysosomal 9kDa, V0 subunit e1 | 5q35.1 | 1.12 | up |
| **5813** | PURA | Purine-rich element binding protein A | 5q31 | 1.11 | up |
| **3902** | LAG3 | Lymphocyte-activation gene 3 | 12p13.32 | 1.16 | up |
| **84705** | GTPBP3 | GTP binding protein 3 (mitochondrial) | 19p13.11 | 1.06 | up |
| **Tumor suppressor** | | | | | |
| **4681** | NBL1 | Neuroblastoma, suppression of tumorigenicity 1 | 1p36.13 | 1.16 | up |
| **7982** | ST7 | Suppression of tumorigenicity 7 | 7q31.2 | 1.11 | up |
| **127262** | TPRG1L | Tumor protein p63 regulated 1-like | 1p36.32 | 1.11 | up |
| **6764** | ST5 | Suppression of tumorigenicity 5 | 11p15 | 1.10 | up |
